# AGHE Faculty Award Presentations and Lectures

**DOI:** 10.1093/geroni/igaf122.396

**Published:** 2025-12-31

**Authors:** Tina Newsham

## Abstract

The AGHE Faculty Award Presentations and Lectures will feature an address by the 2025 Distinguished Faculty Award recipient, Kara Dassell, PhD, FGSA and an address by the 2025 Rising Star Early Career Faculty Award recipient, Angela Person, PhD, JD, MSW, MA. The Distinguished Faculty Award recognizes persons whose teaching stands out as exemplary, innovative, of impact, or any combination thereof. The Rising Star Early-Career Faculty Award acknowledges new faculty whose teaching and leadership stand out as influential and innovative.

